# Reversine attenuates cholestatic ductular reaction in rats

**DOI:** 10.1002/2211-5463.13596

**Published:** 2023-04-07

**Authors:** Di Huang, Lijuan Tang, Tianyang Li, Peilin Li, Zisheng Huang, Jiefeng Weng, Shuai Zhang, Weili Gu, Yu Huang

**Affiliations:** ^1^ Department of Surgery Guangzhou First People's Hospital Guangzhou China; ^2^ Guangzhou Digestive Disease Center Guangzhou First People's Hospital Guangzhou China; ^3^ Department of Emergency The Second Affiliated Hospital of Guangzhou University of Chinese Medicine Guangzhou China

**Keywords:** biliary epithelial cells, cholangiopathy, cholestatic liver disease, ductular reaction, reversine

## Abstract

Ductular reaction (DR) is usually observed in biliary disorders or various liver disorders, including nonalcoholic fatty liver disease. Few studies have focused on interrupting the DR process in the cholestatic environment. Here, we investigated the impact of reversine on DR in rats that had undergone bile duct ligation (BDL). Cholestatic injury was induced in rats 2 weeks following BDL. DR was assessed with biliary markers by immunohistochemistry. Biliary epithelial cells (BECs) were isolated for the analysis of proliferation and biliary factor gene expression. The effects of reversine on DR and fibrosis were analyzed *in vivo* via intraperitoneal injection in rats for 2 weeks. Chemically‐induced BEC formation was used to investigate the biliary markers affected by reversine *in vitro*. DR with increased BEC expansion was identified in cholestatic liver injury, as indicated by CK7, CK19, and EpCAM expression around the portal vein in BDL rats. BDL‐induced DR cells showed the increased expression of genes regulating cell proliferation (*Ki67*, *Foxm1*, and *Pcna*) and biliary markers (*Krt7*, *Krt19*, *Epcam*, *Sox9*, *Cftr*, and *Asbt*). Reversine attenuated cholestatic fibrosis and DR in rats. Reversine affected chemically‐induced BEC formation, with the decreased expression of biliary *Krt7*, *Cftr*, and *Ggt1* genes *in vitro*. BDL‐induced Notch activation was attenuated upon reversine treatment *in vivo*, in part via the Notch/Sox9 pathway. In conclusion, reversine attenuated cholestatic ductular reaction and fibrosis in rats and reduced the bile duct formation associated with Dlk1/Notch/Sox9 signaling. Reversine may be regarded as a potential drug for cholangiopathies for preventing a ductular reaction.

## Graphical abstract

In this study, we demonstrated that reversine attenuates cholestatic ductular reaction and fibrosis in BDL rats and reduces bile duct formation associated with the Dlk1/Notch/Sox9 signaling pathway, suggesting reversine may have the potential for preventing ductular reaction and fibrosis progression in cholangiopathies.

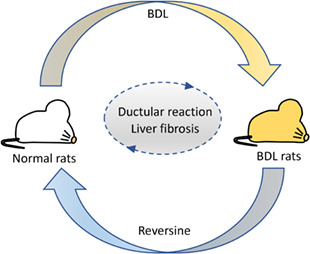

**Abbreviations**
ALP: alkaline phosphatase
BDL: bile duct ligation
BECs: biliary epithelial cells
CLiPs: chemically‐induced liver progenitor cells
DR: ductular reaction
EpCAM: epithelial cell adhesion molecule
HSCs: hepatic stellate cells
T‐Bil: total bilirubin
γ‐GTP: gamma‐glutamyl transferase

Ductular reaction (DR) refers to a process of expansion‐activated biliary epithelial cells (BECs) around the portal vein upon injury, in which a transient luminal epithelium is formed, establishing an auxiliary biliary system [1]. Ductular reaction is usually observed in biliary disorders such as primary biliary cholangitis [2], primary sclerosing cholangitis [3, 4], and biliary atresia [5], or in various liver disorders, including nonalcoholic fatty liver disease [6, 7]. Previous studies showed that expanded BECs during DR transdifferentiated into hepatocytes to support liver regeneration [1, 8]. However, BECs also promote inflammation and peribiliary fibrosis, and the overt proliferation of malignant‐transformed BECs has been found to result in cholangiocarcinoma formation [9, 10]. Few studies have focused on interrupting the DR process in the cholestatic environment. Common bile duct ligation (BDL) causes cholestasis; and a BDL model, therefore, has been used widely to study cholestatic liver injury [11] and fibrogenesis [12]. However, the use of a BDL model for studying DR has seldom been reported.

Reversine, a synthetic 2,6‐disubstituted purine analog, reportedly neutralized liver fibrosis by inducing hepatic stellate cell (HSC) apoptosis, restrained cell proliferation, reduced HSC activation, and degraded the extracellular matrix *in vitro* [13]. Here, we hypothesized that this small molecular compound, reversine, could also attenuate BDL‐induced cholestatic injury by affecting the DR process *in vivo*. Herein, in the present study, we investigated the characteristics of DR caused by BDL and studied the impact of reversine on the BDL‐induced DR process. By applying our previously reported method for BEC conversion for bile duct formation [14] from chemically‐induced liver progenitor cells (CLiPs) [15], we explored the impact of reversine on chemically‐induced BEC formation *in vitro*. The results showed that reversine attenuated cholestatic DR in rats and reduced bile duct formation *in vitro*, in part by downregulating Dlk1/Notch/Sox9 signaling. Reversine may be regarded as a potential drug for cholangiopathies for halting the DR.

## Materials and methods

### Animals

Male Lewis rats aged 7 weeks, weighing between 220 ± 20 g, were purchased from Charles River Company (Beijing, China), and all rats were housed in smooth‐bottomed plastic cages in a pathogen‐free animal room at a controlled temperature (22 ± 2 °C), humidity (50 ± 10%), and light (12 h light–dark cycle) with free access to rodent chow and water. To accustom the animals to the laboratory environment, an acclimation period of 1 week was allowed before the initiation of the experiment. This study was approved by the Institutional Animal Care and Use Committee of Guangzhou First People's Hospital, the Second Affiliated Hospital of South China University of Technology (No: k2022047‐01).

### BDL model

Double ligation of the common bile duct in rats was used as the BDL model [11]. Briefly, the rats were anesthetized with inhaled isoflurane. After an upper midline laparotomy (6 cm), the common bile duct was exposed and ligated twice with non‐absorbable 6‐0 monofilament sutures. The abdomen was closed with 5‐0 braided silk sutures in layers. After 2 weeks, the rats fasted overnight and were sacrificed. Blood samples were collected via the inferior vena cava and centrifuged at 1600 ***g*** for 10 min at 4 °C, and the collected plasma was stored at −80 °C until assayed. Liver tissue samples of the right lobe were collected at 14 days and fixed with a 4% paraformaldehyde phosphate‐buffered solution for histological analysis.

### Reversine administration

Reversine (CAS NO. 656820‐32‐5) was purchased from Sigma‐Aldrich (Shanghai, China) and dissolved in dimethyl sulfoxide according to the manufacturer's instructions. Reversine was intraperitoneally injected at a dose of 200 μg·kg^−1^ body weight at the time of BDL. Then, rats were injected every 3 days for 2 weeks. BDL rats without reversine administration were used as a positive control. The Sham rats served as a normal control group. The rats fasted overnight and then were sacrificed. Blood samples were collected via the inferior vena cava and centrifuged at 1600 ***g*** for 10 min at 4 °C, and the collected plasma was stored at −80 °C until assayed. Liver tissue samples of the right lobe were collected at 14 days and fixed with a 4% paraformaldehyde phosphate‐buffered solution for histological analysis.

### Blood assays

Levels of alkaline phosphatase (ALP), total bilirubin (T‐Bil), and gamma‐glutamyl transferase (γ‐GTP) in the plasma were measured by the laboratory of the Guangzhou First People's Hospital.

### Histopathology

For histological review, the fixed liver tissues were collected and embedded in paraffin. Sections (4 μm) were cut, deparaffinized, and stained with hematoxylin and eosin (HE) and aniline blue (Azan) staining in accordance with standard techniques.

### Immunohistochemistry (IHC)

For immunohistochemistry, paraffin blocks were cut into 4 μm sections, deparaffinized in xylene, and rehydrated in graded ethanol solutions. Endogenous peroxidase activity was blocked with 3% hydrogen peroxide for 20 min, washed with phosphate‐buffered saline (PBS; 0.01 m, pH 7.4) three times for 5 min, and incubated with blocking buffer at 37 °C for 60 min. Tissue sections were incubated overnight at 4 °C with anti‐Desmin (1 : 200, ab15200; Abcam, Guangdong, China), anti‐α‐SMA (1 : 200, ab7817; Abcam), anti‐CK7 (1 : 8000, ab181598; Abcam), anti‐CK19 (1 : 200, ab7755, Abcam), or anti‐EpCAM (1 : 200, ab71916; Abcam) antibodies, and then with horseradish peroxidase (HRP)‐conjugated rabbit anti‐mouse IgG (A9044; 1 : 200) or anti‐rabbit IgG (A0545; 1 : 200) secondary antibodies for 1 h at 37 °C. HRP‐conjugated secondary binding was visualized using a DAB^+^ substrate chromogen system (Dako, Beijing, China). Nuclei were counterstained with hematoxylin. Bright‐field images were captured using an optical microscope (BX53; Olympus, Shanghai, China). Brown staining was considered positive. Positive areas were analyzed by using winroof software (V6.3, Mitani Corporation, Tokyo, Japan). The positive area (%) per high‐power field (200×) was calculated as follows: expression = positive area/total area × 100%.

### Isolation of hepatocytes and biliary cells

Hepatocytes were isolated using a modified two‐step collagenase perfusion [16]. After perfusion with a Ca^2+^‐free Hank's/ethylene glycol tetraacetic acid (EGTA) solution through the portal vein, the liver was perfused with 130 mL of Hank's solution containing 130 unit·mL^−1^ collagenase at 20–30 mL·min^−1^. The liver was extracted and mechanically minced with a surgical knife. The minced liver was then filtered twice with a four‐layer cotton mesh and 45‐μm stainless steel mesh. The suspension was then purified three times in high‐glucose Dulbecco's modified Eagle medium (DMEM) by centrifugation at 50 × ***g*** for 2 min at 4 °C. The cells were resuspended in a 40% Percoll solution (GE Healthcare, Shanghai, China), and the dead cells were removed via centrifugation at 50 × ***g*** for 20 min. All experiments were conducted using purified hepatocytes of at least 90% viability determined using trypan blue. Hepatocytes were plated on collagen‐coated dishes at a density of 6 × 10^4^ cells·cm^−2^, cultured in DMEM/F12 medium containing 2.4 g·L^−1^ NaHCO_3_ and l‐glutamine, and supplemented with 5 mm HEPES, 30 μg·mL^−1^ l‐proline, 0.5 mg·mL^−1^ bovine serum albumin (BSA), 10 ng·mL^−1^ epidermal growth factor, insulin‐transferrin‐serine‐X, 0.1 μm dexamethasone, 10 mm nicotinamide, 1 mm ascorbic acid‐2 phosphate, 100 U·mL^−1^ penicillin, and 100 mg·mL^−1^ streptomycin.

Biliary cell isolation was conducted using a previously described method with modifications [17]. The filtered residue from the hepatocyte isolation was minced into small fragments by using surgical scissors, suspended in BEC digestion medium in a 50‐mL tube, and shaken in a 37 °C water bath for 30 min. The digested tissue was then filtered through a 40‐μm cell strainer. Gradient density centrifugation was used to separate the BECs from non‐parenchymal cell fractions. As reported previously, the best gradient for BEC isolation was about 800 × ***g*** [14, 17], and it was 200 × ***g*** for HSCs, 600 × ***g*** for Kupffer cells, and 900 × ***g*** for sinusoidal endothelial cells [18]. The cells were centrifugated three times at 600 × ***g*** for 15 min to wipe off the HSCs and Kupffer cells. Thereafter, cells were centrifuged three times at 800 × ***g*** for 10 min at 4 °C, washed, and resuspended in 10 mL of 10% fetal bovine serum (FBS)‐DMEM, then centrifuged again at 800 × ***g*** for 5 min at 4 °C. The biliary cells were plated on a collagen‐I‐coated dish containing a BEC culture medium. Compositions of the pre‐perfusion buffer, collagenase buffer, isolation buffer, BEC digestion medium, and BEC culture medium are listed in Table S1.

### Real‐time quantitative polymerase chain reaction (RT‐qPCR)

Cell samples were acquired for mRNA extraction by using spin columns in accordance with the manufacturer's instructions (NucleoSpin RNA II; Macherey‐Nagel, Duren, Germany). cDNA was synthesized from total RNA by using a high‐capacity cDNA reverse transcription kit (Applied Biosystems, Beijing, China). The samples were stored at −30 °C until use. PCR was performed on an Applied Biosystems Step One Plus real‐time PCR system using a TaqMan Gene Expression Assay kit (Applied Biosystems). *Gapdh* was used as the internal reference, and mRNA expression levels were determined using the comparative cycle time (ΔΔ*C*_t_) method [19]. The TaqMan primers used for PCR are listed in Table S2.

### Biliary formation *in vitro*

Biliary epithelial cells and biliary formation were induced *in vitro* as previously reported with chemically‐induced liver progenitor cells [14, 15]. Briefly, 1 day before collecting the CLiPs, commercial mouse embryonic fibroblasts (MEFs; Cat #PMEF‐N, Merck Millipore, Beijing, China) were used to form a MEF feeder layer by seeding 5 × 10^4^ cells on collagen‐coated 12‐well plates (1.3 × 10^4^ cells·cm^−2^) in DMEM containing 10% FBS. Then the dissociated CLiPs were plated onto the MEF feeder layer at a density of 5 × 10^5^ cells per well (1.3 × 10^5^ cells·cm^−2^) in a reprogramming medium supplemented with 5% FBS. Thereafter, the BEC induction medium (BIM) was replaced every 2 days for 6 days, followed by BIM supplemented with 2% growth factor reduced Matrigel (Corning, Beijing, China) for an additional 6 days, to facilitate the maturation of BECs and the formation of the biliary structure. For reversine administration during biliary formation, 0 or 5 μm·mL^−1^ of reversine was added into the medium during the last 6 days, and the medium was changed every 2 days.

### Immunofluorescence staining

Cells were fixed with 4% paraformaldehyde at room temperature (RT) for 10 min, permeabilized in 0.1% Triton X‐100 (Sigma‐Aldrich) in PBS for 10 min, and blocked in PBS containing 1% BSA for 1 h at RT. Then the cells were incubated with primary antibodies, anti‐CK7 (ab181598, 1 : 500), anti‐CFTR (ab2784, 1 : 400), and anti‐α‐tubulin (CST, #12152, 1 : 800) at 4 °C overnight. After washing with PBS twice, the cells were incubated with appropriate secondary antibodies for 2 h at RT. Finally, nuclei were stained with 4′,6‐diamidino‐2‐phenylindole (DAPI, Dojindo, Shanghai, China) for 30 min, and fluorescence images were captured using a confocal laser scanning microscope (FV10i, Olympus).

### Statistical analysis

Data were presented as mean ± standard deviation. Statistical analyses were conducted with graphpad prism (GraphPad Software, Inc., San Diego, CA, USA) using a two‐sided Student's *t* test or ANOVA. A probability (*P*) value < 0.05 was considered statistically significant.

## Results

### Cholestatic liver‐induced ductular reaction in BDL rats

Cholestatic injury was induced after 2 weeks in rats following BDL (A). BDL increased the markers of cholestasis in serum (Fig. S1A), such as T‐Bil, ALP, and γ‐GTP. The injured liver was observed with HE staining (Fig. S1B). The BDL liver showed prominent lobular and portal changes, including periportal and parenchymal fibrillar collagen deposition as shown on Azan staining (Fig. S2A) and inflammatory cell infiltration as evidenced by increasing *Tgfβ1* and *Tnfα* expression (Fig. S2B).

**Fig. 1:**
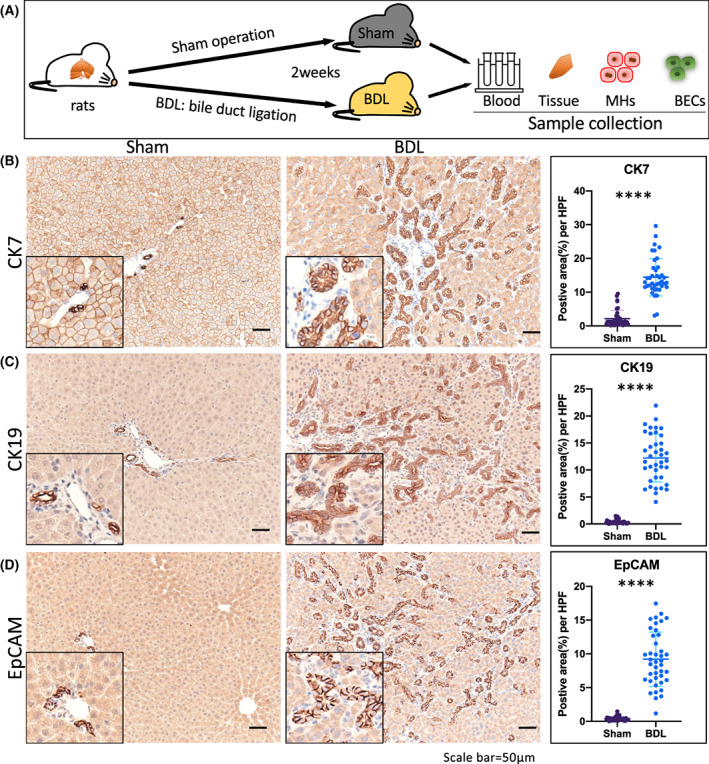
Bile duct ligation (BDL) induced in the cholestatic liver promoted the ductular reaction (DR) in rats. (A) Cholestasis was induced by BDL in rats over the course of 14 days. A sham operation was used as a control. Blood, tissue samples, mature hepatocytes, and biliary epithelial cells were collected for analysis. *n* = 6 per group. Comparative immunostaining images and the positive area per high‐power field of the biliary protein markers CK7 (B) and CK19 (C), and the epithelial cell adhesion molecule, EpCAM (D) in sham and BDL rat livers. Quantitative data indicated the higher expression of CK7 CK19, and EpCAM in BDL livers than in Sham livers. Data were presented as scatter plot with mean ± SD and were compared using the Student's *t* test, ****P < 0.0001. Scale bar = 50 μm.

To measure the DR induced by BDL, the biliary markers, CK7, CK19, and EpCAM, were assessed in the liver tissues. Data showed that BDL injury increased the positive cells marked with CK7 (Fig. 1B), CK19 (Fig. 1C)., and EpCAM (Fig. 1D), around the portal vein, demonstrating the activated BEC expansion. Therefore, 2 weeks of cholestatic injury caused by BDL‐induced DR in rats, characterized as the expansion of activated BECs upon injury.

### BDL‐induced DR cells showed the increased expression of genes regulating proliferation and biliary formation

**Fig. 2:**
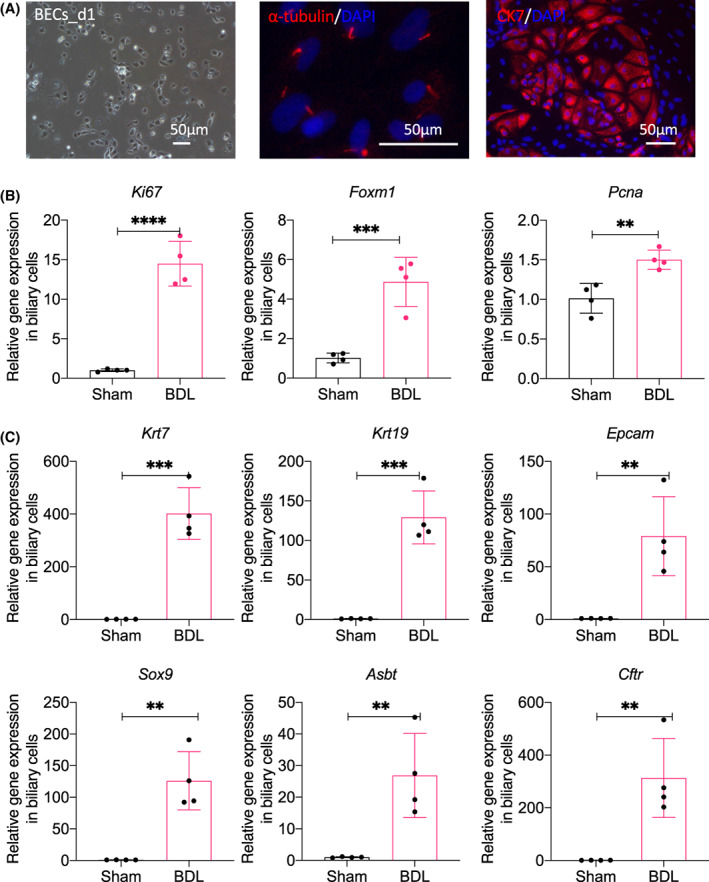
BDL‐induced DR cells showed the increased expression of genes regulating proliferation and biliary markers. (A) Representative image of primary isolated biliary epithelial cells (BECs), and the immunostaining images of the BEC markers α‐tubulin and CK7 (red); the nuclei were stained with DAPI (blue). Scale bar = 50 μm. (B). Levels of the cell proliferation genes *of Ki67*, *Foxm1*, and *Pcna* were analyzed and compared to those of BECs isolated from BDL and Sham rats using RT‐qPCR. Quantitative data showed the increased gene expression of *Ki67*, *Foxm1*, and *Pcna*. (C). Biliary marker genes of *Krt7*, *Krt19*, *Epcam*, *Sox9*, *Asbt*, and *Cftr* were analyzed and compared to BECs isolated from BDL and Sham rats using RT‐qPCR. Quantitative data showed the increased gene expression of *Krt7*, *Krt19*, *Epcam*, *Sox9*, *Asbt*, and *Cftr*.*Gapdh* was used as an internal reference, *n* = 4; Data were presented as scatter plot with mean ± SD and were compared using the Student's *t* test, **P < 0.01, ***P < 0.001, and ****P < 0.0001.

To characterize the genetic expression of the BDL‐induced DR cells, we isolated activated BECs from the BDL rats (Figs 1A and 2A). The isolated cells were positive to α‐tubulin and CK7, showing the biliary‐specific expression of those cells (Fig. 2A). Compared to the sham rats, the BDL‐induced cells showed the increased expression of *Ki67*, *Foxm1*, and *Pcna* genes (Fig. 2B), indicating the increased proliferation‐specific gene expression of the BDL‐induced DR cells. As shown in Fig. 2C, the BDL‐induced DR cells showed increased biliary formation gene expression, such as *Krt7*, *Krt19*, *Epcam*, *Sox9*, *Cftr*, and *Asbt*. These data indicated that the highly proliferative DR cells from BDL could be characterized as being specific to biliary formation.

### Reversine attenuated cholestatic liver fibrosis and ductular reaction in rats

**Fig. 3:**
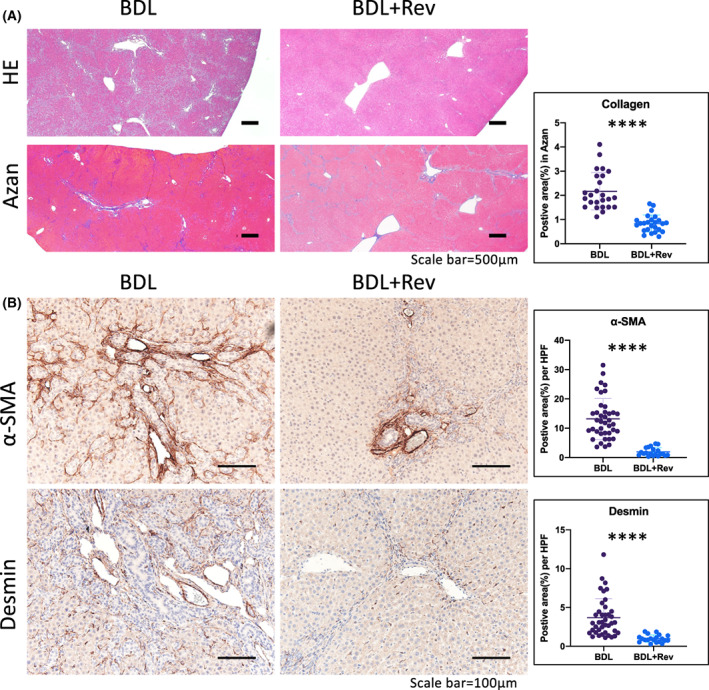
Reversine attenuated the cholestatic liver fibrosis in rats. (A) Representative images of HE and Azan staining in BDL rats with and without reversine administration. Collagen depositions were quantified as the positive areas in Azan stating. Quantitative data showed decreased collagen deposition within reversine treatment. Scale bar = 500 μm. *n* = 6. (B) Comparative immunostaining images and the positive area per high‐power field of the hepatic fibrosis protein markers α‐SMA and Desmin in BDL rats with or without reversine administration. Quantitative data showed decreased α‐SMA and Desmin levels in reversine treatment groups. Data were presented as scatter plot with mean ± SD and were compared using the Student's *t* test, ****P < 0.0001. Scale bar = 100 μm. *n* = 6.

We then studied the impact of reversine on liver fibrosis and the DR in BDL‐induced cholestatic rats. BDL rats given reversine treatment (BDL + Rev) were compared to BDL rats not given reversine treatment (BDL). Serum biochemistry showed the significantly downregulated expression of T‐Bil and γ‐GTP in BDL + Rev (Fig. S1A); Data showed a moderate, but not statistically significant, downregulation of ALP levels upon reversine treatment (Fig. S1A). Histologically, the administration of reversine attenuated liver injury, as shown by HE staining (Fig. 3A), and decreased the fibrillar collagen deposition, as shown by Azan staining (Fig. 3A). We also assessed two markers of liver fibrosis, α‐SMA, and Desmin, via immunostaining in liver tissues (Fig. 3B). The quantitative data showed that reversine significantly decreased the positive areas of α‐SMA, and Desmin staining (Fig. 3B).

**Fig. 4:**
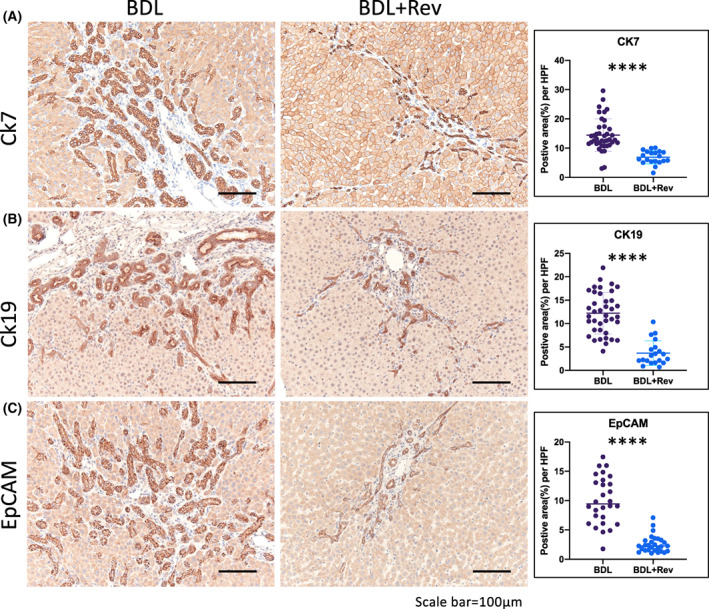
Reversine attenuated the cholestatic ductular reaction in rats. Comparative immunostaining images and the positive area per high‐power field of the biliary markers (A) CK7, (B) CK19, and (C) EpCAM in BDL rats with and without reversine administration. Quantitative data showed decreased CK7, Ck19, and EpCAM expression in reversine treatment groups. Data were presented as scatter plot with mean ± SD and were compared using the Student's *t* test, ****P < 0.0001. Scale bar = 100 μm. *n* = 6.

Because the severity of BDL injury increased BEC expansion marked with CK7‐, CK19‐, and EpCAM‐positive cells (Fig. 1B), we then compared those markers across the sample groups given and not given reversine treatment. The immunostaining and quantitative data showed that levels of the biliary protein markers of CK7 (Fig. 4A), CK19 (Fig. 4B), and EpCAM (Fig. 4C) were significantly decreased upon administration of reversine, demonstrating an attenuated BDL‐induced DR by reversine.

### Reversine affected BEC formation *in vitro*

**Fig. 5:**
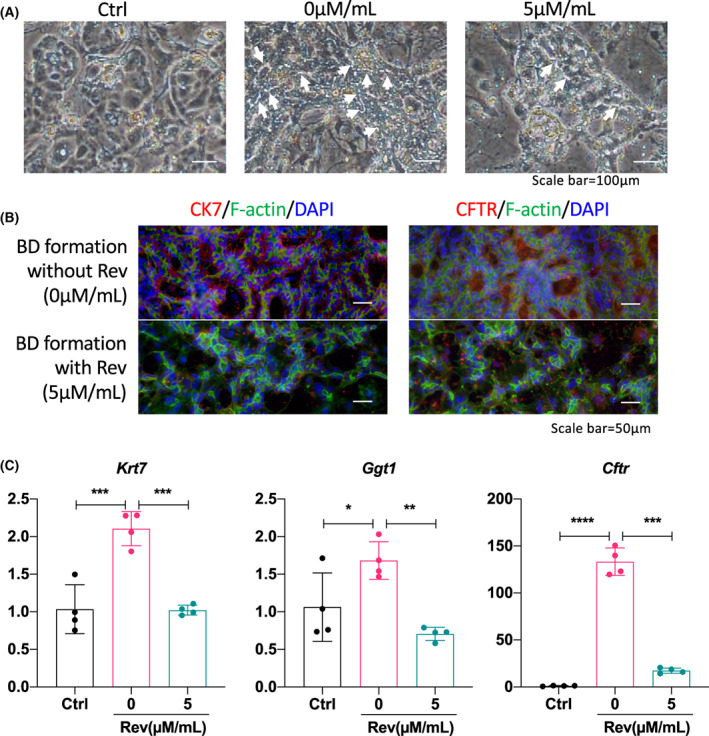
Reversine affected the BEC formation *in vitro*. (A) Images of the chemically‐induced liver progenitor cells before biliary induction (Ctrl) and after biliary induction without or with reversine administration (0 or 5 μm·mL^−1^). The white arrows show the formed ductular formation. Scale bar = 100 μm. *n* = 3. (B) The immunostaining images of BEC protein markers CK7 and CFTR (red) in cells with or without reversine administration. The cytoskeleton was stained with F‐actin (green), and the nuclei were stained with DAPI (blue). Data showed reversine decreased CK7 and CFTR levels during bile duct formation. Scale bar = 50 μm. *n* = 3. (C) The gene expression of the biliary markers *Krt7*, *Krt9*, and *Cftr* were compared and analyzed via RT‐qPCR. Quantitative data showed reversine decreased the gene levels of *Krt7*, *Krt9*, and *Cftr* during bile duct formation. *Gapdh* was used as an internal reference. Data were presented as scatter plot with mean ± SD and were compared using ANOVA, *P < 0.05, **P < 0.01, ***P < 0.001, and ****P < 0.0001. *n* = 3.

Previously, we reported a method for BEC formation from CLiPs (Fig. S3). Here, to investigate the impact of reversine on BEC formation, we applied this *in vitro* method and administered 0 or 5 μm·mL^−1^ of reversine during BEC differentiation and bile duct (BD) formation, compared the cells without biliary formation treatment (termed as Ctrl in figures). As shown in Fig. 5A, the Ctrl cells showed no BEC differentiation or ductulus or biliary formation. Cells that received biliary induction showed several ductular formations (white arrows). However, the administration of reversine during biliary formation showed decreased ductuli (Fig. 5A), indicating that reversine affected BEC differentiation and BD formation. By analyzing the biliary protein markers of CK7 and CFTR, we found that reversine also repressed the expression of both biliary proteins (Fig. 5B). We also found that reversine decreased the BD gene expression of *Krt7*, *Ggt1*, and *Cftr* (Fig. 5C). Those data clearly established that reversine affected BEC formation in the *in vitro* model.

### Reversine affected notch signaling

Previous reports have indicated the importance of Notch signaling in biliary differentiation [20, 21]. We analyzed the gene expression of the known Notch ligands (*Jag1* and *Dlk1*), the receptor (*Notch1*, *Notch2*, *Notch3*, and *Notch4*), and the target genes (*Hes1*, *Hes5*, and *Hey1*). Gene samples were acquired from cells given 0 or 5 μm·mL^−1^ reversine during BEC differentiation and bile duct (BD) formation. Compared to the samples not given reversine treatment (i.e., Rev 0 μm·mL^−1^), data showed the increased gene expression of Notch ligand *Dlk1*, but not the *Jag1*, increased levels of Notch receptors *Notch1* and *Notch2*, and decreased expression of *Notch3* and *Notch4* (Fig. S4). Many of the target genes, such as *Hes5*, though not *Hes1* or *Hey1*, were showed increased expression upon reversine treatment (Fig. S4). These results established that reversine could affect the Notch signaling pathway in our simulated *in vitro* environment.

**Fig. 6:**
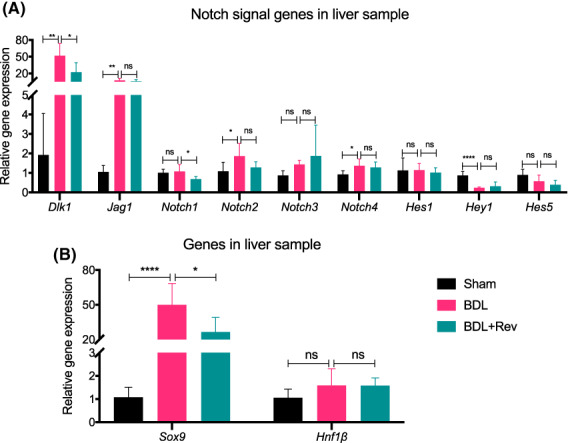
Effect of reversine treatment on the expression of various genes implicated in Notch signaling. (A) The gene expression levels of Notch signaling ligands (*Jag1* and *Dlk1*), receptors (*Notch1*, *Notch2*, *Notch3*, and *Notch4*), and target genes (*Hes1*, *Hes5*, and *Hey1*) were analyzed via RT‐qPCR in *in vivo* liver tissue samples. (B) The gene levels of *Sox9* and *Hnf1*β were analyzed via RT‐qPCR. *Gapdh* as internal reference. *n* = 6; Data were presented as bars with mean ± SD and were compared using ANOVA, *P < 0.05, **P < 0.01, and ****P < 0.0001, ns, no significance.

We then further evaluated the changes in gene expression in the *in vivo* setting utilizing the whole liver samples (Fig. 1A). As shown in Fig. 6A, BDL increased the ligand expression of *Dlk1* and *Jag1*. The levels of the receptors of *Notch2* and *Notch4* significantly increased in BDL liver, and those of *Notch1* and *Notch3* showed a detectable, but not statistically significant, tendency to increase. The target genes of *Hes1* and *Hes5* showed no difference in expression between Sham and BDL liver, though the *Hey1* gene was significantly downregulated upon BDL injury. Compared with the BDL samples, the reversine treatment decreased the level of the Notch ligand *Dlk1*, but it did not affect the ligand *Jag1* (Fig. 6A). Reversine treatment decreased levels of *Notch1*, but it did not affect the levels of receptors *Notch2*, *Notch3* or *Notch4*. However, data showed no significant changes in the expression of the Notch target genes *Hes1*, *Hes5*, or *Hey1* (Fig. 6A). As per previous reports, Notch partially controls the expression of Sox9, a key player in bile system morphogenesis [21]. We further analyzed the expression of Sox9 as well as Hnf1β, which is a necessary factor in biliary epithelium formation from the onset of the development of the biliary system [22]. The significantly increased levels of *Sox9* in BDL liver became attenuated upon reversine treatment (Fig. 6B). However, the increases in *Hnf1β* levels appeared unaffected by reversine in BDL livers (Fig. 6B). Τhese findings showed that the impact of reversine on livers *in vivo* is associated with the Dlk1/Notch/Sox9 pathway.

## Discussion

Ductular reaction demonstrates histological, cellular, molecular, and tissue diversity, which are believed to cause the diverse outcomes of DR of hepatobiliary regeneration, fibrogenesis, and hepatocarcinogenesis [23]. In the model of BDL here, which caused a cholestatic internal environment, both biliary proliferation and fibrogenesis were present. Thus, DR behaves as a double‐edged sword [24]. Indeed, as reported previously, expanded DR cells transdifferentiated into hepatocytes to support liver regeneration [1, 8]. Conversely, increased DR also promoted inflammation and peribiliary fibrosis, and overt proliferation of malignant transformed BECs resulted in cholangiocarcinoma formation [9, 10]. It should be clear to investigators that focusing on attenuating the DR process may advance our understanding of developing novel therapeutic strategies for curing cholangiopathies.

In this study, we first investigated the details of DR caused by BDL. Data showed that BDL injury increased the cells positive for the markers CK7 and CK19 around the portal vein, demonstrating BEC expansion and the formation of a biliary system. Furthermore, we also found increased EpCAM in the luminal epitheliums forming the biliary system. Previous research demonstrated that EpCAM was highly expressed in hepatic stem cells or progenitor cells, and was absent in mature hepatocytes [25, 26, 27]. Consistent with these previous studies, EpCAM was associated with the maintenance of the undifferentiated phenotype of embryonic stem cells [28, 29]. Therefore, the EpCAM^+^ cell has been regarded as a potential hepatic stem cell residing in the normal and injured liver. From this point of view, in our study, the DR cell was an EpCAM^+^ cell, demonstrating the stem cell properties of the DR cells. This may be one of the reasons for DR cell trans‐differentiation into hepatocytes to support liver regeneration under certain conditions. Genetically, we found that isolated BECs showed the increased expression of genes regulating cell proliferation, such as *Ki67*, *Foxm1*, and *Pcna*, and increased biliary markers, such as *Krt7*, *Krt19*, *Epcam*, *Sox9*, *Cftr*, and *Asbt*, indicating cell proliferation and biliary formation in DR cells. However, fibrogenesis also accompanied the DR process. Furthermore, we found that isolated BECs showed increased gene expression levels of the pan‐HSC marker Desmin and the Kupffer cell marker CD45 (Fig. S5A,B), but there were no significant changes in the expression of the CD31 gene (Fig. S5C) between the BDL and Sham groups.

Previously, we studied the effects of reversine on the cell cycle, apoptosis, and activation of HSCs *in vitro* [13] and on liver injury and hepatic fibrosis *in vivo*. We found that reversine neutralized hepatic fibrosis by inducing HSC apoptosis, restrained cell proliferation, reduced HSCs activation, and degraded the extracellular matrix [13]. With the hypothesis that reversine attenuated BDL‐induced cholestatic injury, we studied the effects of reversine on the BDL‐induced DR process. Our results supported this hypothesis by both *in vivo* and *in vitro* results. In the *in vivo* study, serum biochemical data showed restoration of the T‐Bil and γ‐GTP levels. Histologically, reversine significantly decreased the expression of the liver fibrosis markers α‐SMA and Desmin, and decreased the expression of biliary protein markers CK7, CK19, and EpCAM, demonstrating reversine's ability to attenuate DR and fibrogenesis. In the *in vitro* study, we explored the impact of reversine on bile duct formation by applying the previously reported method for bile duct formation [14]. Upon biliary induction, the cells showed several ductular formations, while the administration of reversine showed decreased ductuli during biliary formation, indicating that reversine reduced bile duct formation *in vitro*.

Notch signaling has been shown to be important in biliary differentiation [20, 21, 30]. A previous report showed that inhibition of this signaling pathway reduced the differentiation of hepatic progenitor cells into cholangiocytes in biliary atresia [31]. The impairment of Notch signaling was reported to associate with Alagille syndrome, in which individuals do not have enough bile ducts [32]. By analyzing the Notch signal genes *in vitro* cell samples and in *in vivo* liver tissue samples, we found that the trends in changes in Notch ligand and receptor levels differed between those two systems. These trends may have been caused by the differences between the *in vivo* model and the *in vitro* system. In our *in vitro* system, we used CLiPs, which are chemically‐induced liver progenitor cells [33, 34], for bile duct formation [35]. As per our results, BDL injury caused increases in the levels of the ligands *Jag1* and *Dlk1* in the liver. Jag1, a canonical Notch ligand, functioned as an activator of Notch receptors on adjacent cells. Dlk1, a non‐canonical Notch ligand, is a member of the epidermal growth factor‐like repeat‐containing family of proteins [36]. Increasing evidence indicates that Dlk1 functions as a secreted or transmembrane protein, and interacted with Notch to act as a negative regulator of Notch activation and signaling [37, 38]. Dlk1 was also found to be a potentiator of adipogenesis of mesenchymal cells. It functioned as a Notch signaling inhibitor [37]. The simultaneous increases in *Jag1* and *Dlk1* indicated that the cholestatic environment plays a complicated role in BDL rats. In our results, the levels of the receptors *Notch2* and *Notch4* significantly increased in BDL livers. *Notch1* and *Notch3* levels showed a detectable but not statistically significant tendency to increase. These changes in Notch signals may be attributable to BDL‐induced DR, which acts as a double‐edged sword. With reversine treatment, the levels of the ligand *Dlk1* decreased, followed by a decrease in that of *Notch1* receptor. Our data, however, showed no statistically significant changes in levels of the Notch target genes of *Hes1*, *Hes5*, or *Hey1*, indicating the involvement of some other target gene during reversine treatment. Previous reports showed Sox9 to be a Notch target regulating morphogenesis in the bile system [21]. Our data indicated that reversine significantly neutralized the elevation of Sox9 expression caused by BDL, indicating the attenuation of biliary formation by reversine, which was regulated in part via Notch/Sox9 signaling *in vivo*. In other words, reversine may be a potential drug to attenuate biliary formation associated with Dlk1/Notch/Sox9 signaling. The mode of action of reversine still remains unclear, so it should be further explored in the future.

A major limitation of our *in vitro* studies relates to the isolation of the primary BECs for gene analysis. Although we used gradient density centrifugation to separate the BECs from non‐parenchymal cell fractions, we still observed HSC, Kupffer cell, and endothelial cell populations in our isolated BEC samples. Such contamination may have been avoided through the use of magnetic‐activated cell sorting or fluorescence‐activated cell sorting. However, HSCs and Kupffer cells were present in both the Sham and BDL groups at the same level, which was acceptable in our setting.

In conclusion, reversine attenuated cholestatic DR and fibrosis in rats and reduced bile duct formation associated with Dlk1/Notch/Sos9 signaling. Our study helps to advance our understanding of BEC biology in BDL‐induced DR cells and the development of novel therapeutic strategies to treat cholangiopathies. Reversine may be regarded as a potential drug for cholangiopathies for halting the ductular reaction.

## Conflict of interest

The authors declare no conflict of interest.

## Author contributions

YH and WG conceived and designed the research. DH and LT performed experiments. TL, PL, and ZH analyzed the data. SZ and JW prepared the figs YH edited and revised the article. YH and WG approved the final version of the article.

## Supporting information


**Fig. S1.** BDL‐induced cholestatic injury in liver. (A) The serum level of T‐Bil, γ‐GTP, and ALP in the Sham group, the BDL, and the BDL + Rev rats, *n* = 6 per group. Data were presented as scatter plot with mean ± SD and were compared using ANOVA, *P < 0.05, **P < 0.01, and ***P < 0.001, ns—no significance. (B) Representative images of HE stains in the Sham group, BDL, and BDL + Rev liver tissue. Scale bar = 500 μm.
**Fig. S2.** BDL‐induced collagen deposition and inflammatory factors in liver. (A) Images of Azan staining in Sham and BDL rats. Collagen deposition was quantified via positive areas in Azan staining. ****P < 0.0001. Scale bar = 500 μm. *n* = 6. (B) The gene expression of inflammatory factors of *Sox9* and *Hnf1β* were compared and analyzed via RT‐qPCR in Sham and BDL livers. *Gapdh* served as an internal reference. Data were presented as scatter plot with mean ± SD and were compared using the Student's *t* test, **P < 0.01 and ****P < 0.0001.
**Fig. S3.** Biliary cells and bile duct (BD) induction method. (A) The schematic of the BD induction from chemically‐induced liver progenitor cells (CLiPs). Refer to our previous study for detailed steps and information (PMID: 33811654; PMID: 33378128). (B) Representative images of cells before and after induction. The arrows show the induced BD structures. Scale bar = 100 μm.
**Fig. S4.** Reversine affected the Notch signal *in vitro*. The gene expression levels of Notch signaling ligands (*Jag1* and *Dlk1*), receptors (*Notch1*, *Notch2*, *Notch3*, and *Notch4*), and the target genes (*Hes1*, *Hes5*, and *Hey1*) were analyzed via RT‐qPCR in the *in vitro* cell samples. *Gapdh* served as an internal reference. *n* = 4; Data were presented as bars with mean ± SD and were compared using ANOVA, *P < 0.05, ***P < 0.001, ****P < 0.0001, ns—no significance.
**Fig. S5.** Gene marker of nonparenchymal cells. (A) The gene expression of HSC of *Desmin* was compared and analyzed via RT‐qPCR in BEC samples isolated from the Sham and BDL group. (B) The gene expression of Kupffer cell of *CD45* was compared and analyzed via RT‐qPCR in BEC samples isolated from the Sham and BDL group. (C) The gene expression of the endothelial cell of *CD31* was compared and analyzed via RT‐qPCR in BEC samples isolated from the Sham and BDL group. *Gapdh* served as an internal reference. Data were presented as scatter plot and were compared using the Student's *t* test, ****P < 0.0001, ns—no significance.
**Table S1.** List of key reagents and resources used in Methods.
**Table S2.** List of TaqMan primers used for qRT‐PCR.

## Data Availability

The data that support the findings of this study are available from the corresponding author (eyhy@scut.edu.cn) upon reasonable request.

## References

[1] Kamimoto K , Kaneko K , Kok CY‐Y , Okada H , Miyajima A and Itoh T (2016) Heterogeneity and stochastic growth regulation of biliary epithelial cells dictate dynamic epithelial tissue remodeling. Elife 5, 1–26. 10.7554/eLife.15034 PMC4951195 27431614

[2] Overi D , Carpino G , Cristoferi L , Onori P , Kennedy L , Francis H , Zucchini N , Rigamonti C , Viganò M , Floreani A *et al*. (2022) Role of ductular reaction and ductular‐canalicular junctions in identifying severe primary biliary cholangitis. JHEP Rep 4, 100556. 36267871 10.1016/j.jhepr.2022.100556 PMC9576897

[3] Carpino G , Cardinale V , Folseraas T , Overi D , Floreani A , Franchitto A , Onori P , Cazzagon N , Berloco PB , Karlsen TH *et al*. (2018) Hepatic stem/progenitor cell activation differs between primary Sclerosing and primary biliary cholangitis. Am J Pathol 188, 627–639. 29248458 10.1016/j.ajpath.2017.11.010

[4] Lewis J (2017) Pathological patterns of biliary disease. Clin Liver Dis 10, 107–110. 10.1002/cld.667 PMC6467117 30992767

[5] Zagory JA , Dietz W , Park A , Fenlon M , Xu J , Utley S , Mavila N and Wang KS (2017) Notch signaling promotes ductular reactions in biliary atresia. J Surg Res 215, 250–256. 28688656 10.1016/j.jss.2017.03.051

[6] Chen Y , Gao W‐K , Shu Y‐Y and Ye J (2022) Mechanisms of ductular reaction in non‐alcoholic steatohepatitis. World J Gastroenterol 28, 2088–2099. 35664038 10.3748/wjg.v28.i19.2088 PMC9134136

[7] Sato K , Marzioni M , Meng F , Francis H , Glaser S and Alpini G (2019) Ductular reaction in liver diseases: pathological mechanisms and translational significances. Hepatology 69, 420–430. 30070383 10.1002/hep.30150 PMC6324973

[8] Raven A , Lu W‐Y , Man TY , Ferreira‐Gonzalez S , O'Duibhir E , Dwyer BJ , Thomson JP , Meehan RR , Bogorad R , Koteliansky V *et al*. (2017) Cholangiocytes act as facultative liver stem cells during impaired hepatocyte regeneration. Nature 547, 350–354. 28700576 10.1038/nature23015 PMC5522613

[9] Karlsen TH , Folseraas T , Thorburn D and Vesterhus M (2017) Primary sclerosing cholangitis – a comprehensive review. J Hepatol 67, 1298–1323. 28802875 10.1016/j.jhep.2017.07.022

[10] Lazaridis KN , Strazzabosco M and LaRusso NF (2004) The cholangiopathies: disorders of biliary epithelia. Gastroenterology 127, 1565–1577. 15521023 10.1053/j.gastro.2004.08.006

[11] Zhang S , Huang D , Weng J , Huang Y , Liu S , Zhang Q , Li N , Wen M , Zhu G , Lin F *et al*. (2016) Neutralization of Interleukin‐17 attenuates Cholestatic liver fibrosis in mice. Scand J Immunol 83, 102–108. 26484852 10.1111/sji.12395

[12] Zhao S , Li N , Zhen Y , Ge M , Li Y , Yu B , He H and Shao R (2015) Protective effect of gastrodin on bile duct ligation‐induced hepatic fibrosis in rats. Food Chem Toxicol 86, 202–207. 26498411 10.1016/j.fct.2015.10.010

[13] Huang Y , Huang D , Weng J , Zhang S , Zhang Q , Mai Z and Gu W (2016) Effect of reversine on cell cycle, apoptosis, and activation of hepatic stellate cells. Mol Cell Biochem 423, 9–20. 27734224 10.1007/s11010-016-2815-x

[14] Huang Y , Sakai Y , Hara T , Katsuda T , Ochiya T , Gu WL , Miyamoto D , Hamada T , Hidaka M , Kanetaka K *et al*. (2021) Bioengineering of a CLiP‐derived tubular biliary‐duct‐like structure for bile transport *in vitro* . Biotechnol Bioeng 118, 2572–2584. 33811654 10.1002/bit.27773

[15] Huang Y , Miyamoto D , Li PL , Sakai Y , Hara T , Adachi T , Soyama A , Hidaka M , Kanetaka K , Gu WL *et al*. (2021) Chemical conversion of aged hepatocytes into bipotent liver progenitor cells. Hepatol Res 51, 323–335. 33378128 10.1111/hepr.13609

[16] Seglen PO (1976) Preparation of isolated rat liver cells. Methods Cell Biol 13, 29–83. 177845 10.1016/s0091-679x(08)61797-5

[17] Katsuda T , Ochiya T and Sakai Y (2019) Generation of hepatic organoids with biliary structures. Methods Mol Biol 1905, 175–185. 30536100 10.1007/978-1-4939-8961-4_16

[18] Bale SS , Geerts S , Jindal R and Yarmush ML (2016) Isolation and co‐culture of rat parenchymal and non‐parenchymal liver cells to evaluate cellular interactions and response. Sci Rep 6, 1–10. 27142224 10.1038/srep25329 PMC4855170

[19] Schmittgen TD and Livak KJ (2008) Analyzing real‐time PCR data by the comparative CT method. Nat Protoc 3, 1101–1108. 18546601 10.1038/nprot.2008.73

[20] Morell CM and Strazzabosco M (2014) Notch signaling and new therapeutic options in liver disease. J Hepatol 60, 885–890. 24308992 10.1016/j.jhep.2013.11.028

[21] Zong Y , Panikkar A , Xu J , Antoniou A , Raynaud P , Lemaigre F and Stanger BZ (2009) Notch signaling controls liver development by regulating biliary differentiation. Development 136, 1727–1739. 19369401 10.1242/dev.029140 PMC2673761

[22] Coffinier C , Gresh L , Fiette L , Tronche F , Schütz G , Babinet C , Pontoglio M , Yaniv M and Barra J (2002) Bile system morphogenesis defects and liver dysfunction upon targeted deletion of HNF1β. Development 129, 1829–1838. 11934849 10.1242/dev.129.8.1829

[23] Gouw ASH , Clouston AD and Theise ND (2011) Ductular reactions in human liver: diversity at the interface. Hepatology 54, 1853–1863. 21983984 10.1002/hep.24613

[24] Sun T , Annunziato S and Tchorz JS (2019) Hepatic ductular reaction: a double‐edged sword. Aging (Albany NY) 11, 9223–9224. 31645478 10.18632/aging.102386 PMC6874463

[25] de Boer CJ , van Krieken JHJM , Janssen‐van Rhijn CM and Litvinov SV (1999) Expression of ep‐CAM in normal, regenerating, metaplastic, and neoplastic liver. J Pathol 188, 201–206. 10398165 10.1002/(SICI)1096-9896(199906)188:2<201::AID-PATH339>3.0.CO;2-8

[26] Schmelzer E , Wauthier E and Reid LM (2006) The phenotypes of pluripotent human hepatic progenitors. Stem Cells 24, 1852–1858. 16627685 10.1634/stemcells.2006-0036

[27] Zhang L , Theise N , Chua M and Reid LM (2008) The stem cell niche of human livers: symmetry between development and regeneration. Hepatology 48, 1598–1607. 18972441 10.1002/hep.22516

[28] González B , Denzel S , Mack B , Conrad M and Gires O (2009) EpCAM is involved in maintenance of the murine embryonic stem cell phenotype. Stem Cells 27, 1782–1791. 19544432 10.1002/stem.97

[29] Lu T‐Y , Lu R‐M , Liao M‐Y , Yu J , Chung C‐H , Kao C‐F and Wu H‐C (2010) Epithelial cell adhesion molecule regulation is associated with the maintenance of the undifferentiated phenotype of human embryonic stem cells. J Biol Chem 285, 8719–8732. 20064925 10.1074/jbc.M109.077081 PMC2838295

[30] Kodama Y , Hijikata M , Kageyama R , Shimotohno K and Chiba T (2004) The role of notch signaling in the development of intrahepatic bile ducts. Gastroenterology 127, 1775–1786. 15578515 10.1053/j.gastro.2004.09.004

[31] Mao Y , Tang S , Yang L and Li K (2018) Inhibition of the notch signaling pathway reduces the differentiation of hepatic progenitor cells into Cholangiocytes in biliary atresia. Cell Physiol Biochem 49, 1115–1123. 10.1159/000493290 30196281

[32] McDaniell R , Warthen DM , Sanchez‐Lara PA , Pai A , Krantz ID , Piccoli DA and Spinner NB (2006) NOTCH2 mutations cause Alagille syndrome, a heterogeneous disorder of the NOTCH signaling pathway. Am J Hum Genet 79, 169–173. 16773578 10.1086/505332 PMC1474136

[33] Katsuda T , Kawamata M , Hagiwara K , Takahashi RU , Yamamoto Y , Camargo FD and Ochiya T (2017) Conversion of terminally committed hepatocytes to Culturable Bipotent progenitor cells with regenerative capacity. Cell Stem Cell 20, 41–55. 27840021 10.1016/j.stem.2016.10.007

[34] Katsuda T and Ochiya T (2019) Chemically induced liver progenitors (CLiPs): a novel cell source for hepatocytes and biliary epithelial cells. Methods Mol Biol 1905, 117–130. 30536095 10.1007/978-1-4939-8961-4_11

[35] Huang Y , Sakai Y , Hara T , Katsuda T , Ochiya T , Gu WL , Miyamoto D , Hamada T , Kanetaka K , Adachi T *et al*. (2020) Differentiation of chemically induced liver progenitor cells to cholangiocytes: investigation of the optimal conditions. J Biosci Bioeng 130, 545–552. 32782195 10.1016/j.jbiosc.2020.07.009

[36] Grassi ES and Pietras A (2022) Emerging roles of DLK1 in the stem cell niche and cancer Stemness. J Histochem Cytochem 70, 17–28. 34606325 10.1369/00221554211048951 PMC8721543

[37] Nueda M‐L , Baladrón V , Sánchez‐Solana B , Ballesteros M‐Á and Laborda J (2007) The EGF‐like protein dlk1 inhibits notch signaling and potentiates Adipogenesis of mesenchymal cells. J Mol Biol 367, 1281–1293. 17320900 10.1016/j.jmb.2006.10.043

[38] Bray SJ , Takada S , Harrison E , Shen S‐C and Ferguson‐Smith AC (2008) The atypical mammalian ligand Delta‐like homologue 1 (Dlk1) can regulate notch signalling in Drosophila. BMC Dev Biol 8, 11. 18237417 10.1186/1471-213X-8-11 PMC2268666

