# Supplementary material for: Reversine attenuates cholestatic ductular reaction in rats
Source: FEBS Open Bio. 2023 Apr 7;13(5):898–911. doi: 10.1002/2211-5463.13596 (PMC10153338; doi:10.1002/2211-5463.13596)
Supplement: Supplementary file 1 — Fig. S1. BDL‐induced cholestatic injury in liver. (A) The serum level of T‐Bil, γ‐GTP, and ALP in the Sham group, the BDL, and the BDL + Rev rats, n = 6 per group. Data were presented as scatter plot with mean ± SD and were compared using ANOVA, *P < 0.05, **P < 0.01, and ***P < 0.001, ns—no significance. (B) Representative images of HE stains in the Sham group, BDL, and BDL + Rev liver tissue. Scale bar = 500 μm. Fig. S2. BDL‐induced collagen deposition and inflammatory factors in liver. (A) Images of Azan staining in Sham and BDL rats. Collagen deposition was quantified via positive areas in Azan staining. ****P < 0.0001. Scale bar = 500 μm. n = 6. (B) The gene expression of inflammatory factors of Sox9 and Hnf1β were compared and analyzed via RT‐qPCR in Sham and BDL livers. Gapdh served as an internal reference. Data were presented as scatter plot with mean ± SD and were compared using the Student's t test, **P < 0.01 and ****P < 0.0001. Fig. S3. Biliary cells and bile duct (BD) induction method. (A) The schematic of the BD induction from chemically‐induced liver progenitor cells (CLiPs). Refer to our previous study for detailed steps and information (PMID: 33811654; PMID: 33378128). (B) Representative images of cells before and after induction. The arrows show the induced BD structures. Scale bar = 100 μm. Fig. S4. Reversine affected the Notch signal in vitro. The gene expression levels of Notch signaling ligands (Jag1 and Dlk1), receptors (Notch1, Notch2, Notch3, and Notch4), and the target genes (Hes1, Hes5, and Hey1) were analyzed via RT‐qPCR in the in vitro cell samples. Gapdh served as an internal reference. n = 4; Data were presented as bars with mean ± SD and were compared using ANOVA, *P < 0.05, ***P < 0.001, ****P < 0.0001, ns—no significance. Fig. S5. Gene marker of nonparenchymal cells. (A) The gene expression of HSC of Desmin was compared and analyzed via RT‐qPCR in BEC samples isolated from the Sham and BDL group. (B) The gene expression of Kupffer [file FEB4-13-898-s001.docx]

**Supplementary Figures**

**
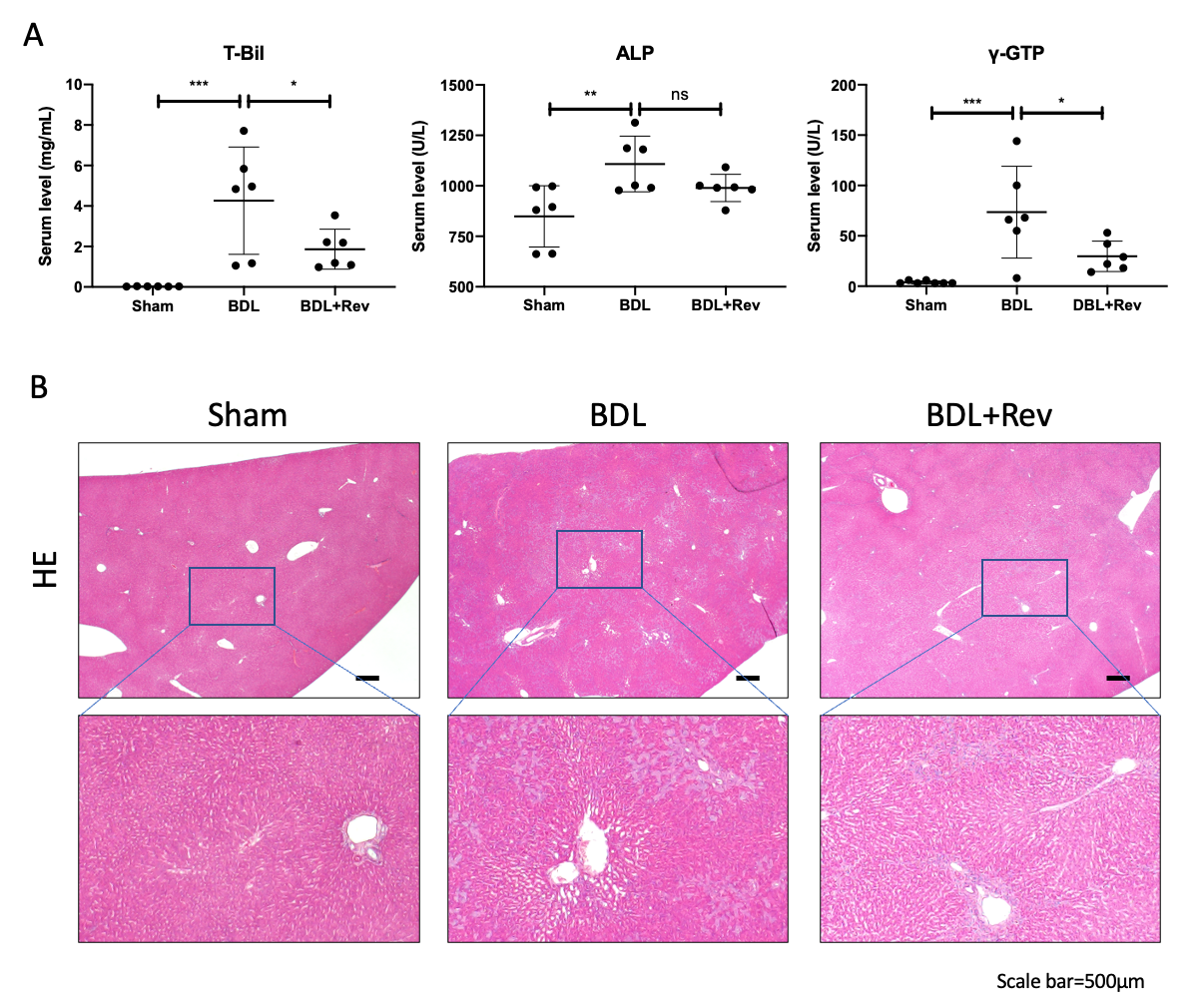
**

**Figure S1.** BDL-induced cholestatic injury in liver. (A) The serum level of T-Bil, γ-GTP and ALP in the Sham group, the BDL and the BDL+Rev rats, n=6 per group. Data were presented as scatter plot with means ± SD and were compared using ANOVA, **P*<0.05, ***P*<0.01, and ****P*<0.001, ns: no significance. (B) Representative images of HE stains in Sham, BDL and BDL+Rev liver tissue. Scale bar = 500μm.


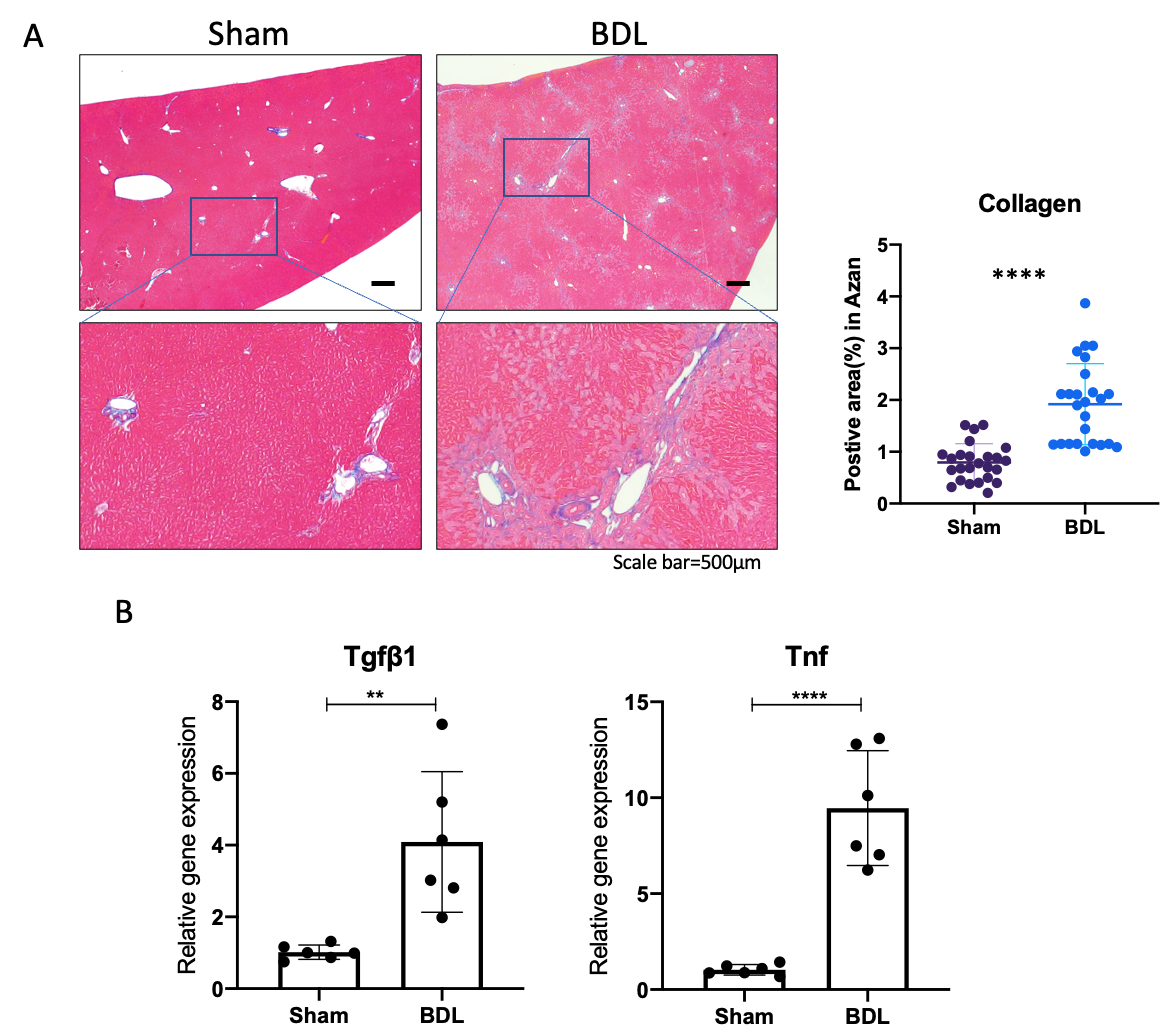


**Figure S2.** BDL-induced collagen deposition and inflammatory factors in liver. (A) Images of Azan staining in Sham and BDL rats. Collagen deposition was quantified via positive areas in Azan staining. *****P*<0.0001. Scale bar = 500μm. n=6. (B) The gene expression of inflammatory factors of *Sox9* and *Hnf1β* were compared and analyzed via RT-qPCR in Sham and BDL livers. *Gapdh* served as an internal reference. Data were presented as scatter plot with means ± SD and were compared using Student’s t test, ***P*<0.01and *****P*<0.0001.


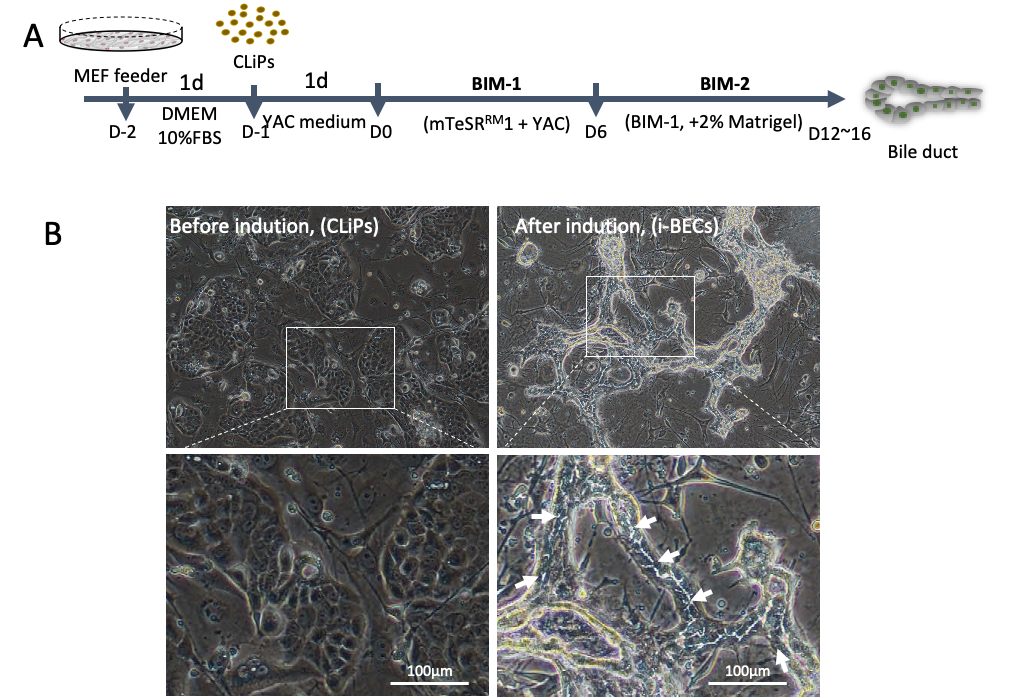


**Figure S3.** Biliary cells and bile duct (BD) induction method. (A) The schematic of the BD induction from chemically-induced liver progenitor cells (CLiPs). Refer to our previous study for detailed steps and information (PMID: 33811654; PMID: 33378128). (B) Representative images of cells before and after induction. The arrows show the induced BD structures. Scale bar = 100μm.

**Figure S4.** Reversine affected the Notch signal *in vitro*. The gene expression levels of Notch signaling ligands (*Jag1* and *Dlk1*), receptors (*Notch1, Notch2, Notch3*, and *Notch4*) and the target genes (*Hes1, Hes5* and *Hey1*) were analyzed via RT-qPCR in the *in vitro* cell samples. *Gapdh* served as an internal reference. n=4; Data were presented as bars with means ± SD and were compared using ANOVA, **P*<0.05, ****P*<0.001, *****P*<0.0001, ns: no significance.

**Figure S5.** Gene marker of non-parenchymal cells. (A) The gene expression of HSC of *Desmin* was compared and analyzed via RT-qPCR in BEC samples isolated from Sham and BDL group. (B) The gene expression of Kupffer cell of *CD45* was compared and analyzed via RT-qPCR in BEC samples isolated from Sham and BDL group. (C) The gene expression of endothelial cell of *CD31* was compared and analyzed via RT-qPCR in BEC samples isolated from Sham and BDL group. *Gapdh* served as an internal reference. Data were presented as scatter plot and were compared using Student’s t test, *****P*<0.0001, ns: no significance.

**Table S1. List of key reagents and resources used in Methods**

| Reagent or Resource | Abbreviation | Concentration | Source |
| --- | --- | --- | --- |
| Pre-perfusion buffer | | | |
| Hanks' balanced salt solution, without calcium chloride and magnesium sulfate | HBSS, (-) | - | [Sigma, H9394](https://www.sigmaaldrich.com/catalog/product/sigma/h9394?lang=ja&region=JP) |
| Ethylene glycol-bis(β-aminoethyl ether)-N,N,N′,N′-tetraacetic acid | EGTA | 5 mM | [Wako Pure Chemical Industries, 346-01312](https://labchem-wako.fujifilm.com/jp/product/detail/W01T02G002.html) |
| Gibco™ HEPES (1M) | HEPES | 10 mM | [Gibco™ 15630080](https://www.fishersci.com/shop/products/gibco-hepes-1m-3/15630080) |
| Gibco™ Penicillin-Streptomycin-Glutamine (100X) | PSG | 1x | [Gibco™ 10378016](https://www.fishersci.com/shop/products/gibco-penicillin-streptomycin-glutamine-100x/10378016#?keyword=Gibco+10378016) |
| Collagenase buffer | | | |
| Hanks' balanced salt solution, with calcium chloride and magnesium sulfate | HBSS, (+) |  | [Sigma, h9269](https://www.sigmaaldrich.com/catalog/product/sigma/h9269?lang=ja&region=JP) |
| Gibco™ HEPES (1M) | HEPES | 10 mM | [Gibco™ 15630080](https://www.fishersci.com/shop/products/gibco-hepes-1m-3/15630080) |
| Gibco™ Penicillin-Streptomycin-Glutamine (100X) | PSG | 1x | [Gibco™ 10378016](https://www.fishersci.com/shop/products/gibco-penicillin-streptomycin-glutamine-100x/10378016#?keyword=Gibco+10378016) |
| GLUCOSE INJECTION 50% | 50% GLU | 0.16% | [Otuska Pharmaceutical Factory](https://www.otsukakj.jp/med_nutrition/dikj/menu1/000232.php) |
| MEYLON Injection 7% | NaHCO3 | 2.50% | [Otsuka Pharmaceutical Factory](https://www.otsukakj.jp/med_nutrition/dikj/menu1/000296.php) |
| Collagenase | - | 0.5 mg/mL | [Wako Pure Chemical Industries, 032-22364](https://labchem-wako.fujifilm.com/jp/product/detail/034-22363.html) |
| Trypsin inhibitor | TI | 0.1 mg/mL | [Wako Pure Chemical Industries, 204-09225](https://labchem-wako.fujifilm.com/jp/product/detail/W01W0120-0922.html) |
| Isolation buffer | | | |
| D-MEM low glusoce | DMEM-LG | - | [Wako Pure Chemical Industries, 041-29775](https://labchem-wako.fujifilm.com/jp/product/detail/W01W0104-2977.html) |
| 350 g/L Glucose stock solution | - | 3.5 g/L | home-made |
| Gibco™ HEPES (1M) | HEPES | 10 mM | [Gibco™ 15630080](https://www.fishersci.com/shop/products/gibco-hepes-1m-3/15630080) |
| Gibco™ Penicillin-Streptomycin-Glutamine (100X) | PSG | 1x | [Gibco™ 10378016](https://www.fishersci.com/shop/products/gibco-penicillin-streptomycin-glutamine-100x/10378016#?keyword=Gibco+10378016) |
| Gibco™ Fetal Bovine Serum | FBS | 10% | [Gibco™ 26140079](https://www.fishersci.com/shop/products/gibco-fetal-bovine-serum-qualified-us-origin-standard-sterile-filtered-3/26140079#?keyword=Gibco+26140-079) |
| DMEM medium | | | |
| D-MEM (Low Glucose) with L-Glutamine and Phenol Red | D-MEM | - | [Wako Pure Chemical Industries, 041-29775](https://labchem-wako.fujifilm.com/jp/product/detail/W01W0104-2977.html) |
| Gibco™ Fetal Bovine Serum | FBS | 10% | [Gibco™ 26140079](https://www.fishersci.com/shop/products/gibco-fetal-bovine-serum-qualified-us-origin-standard-sterile-filtered-3/26140079#?keyword=Gibco+26140-079) |
| Gibco™ Penicillin-Streptomycin-Glutamine (100X) | PSG | 1x | [Gibco™ 10378016](https://www.fishersci.com/shop/products/gibco-penicillin-streptomycin-glutamine-100x/10378016#?keyword=Gibco+10378016) |
| 350 g/L Glucose stock solution | - | 3.5 g/L | home-made |
| BEC-induction medium. (BIM) | | | |
| mTeSR ^TM^ 1 Complete Kit | - | - | [STEMCELL Technologies, #85850](https://www.stemcell.com/mtesr1.html) |
| Y-27632 2HCl | Y | 10 uM | [AdooQ BioScience, A11001-50](https://www.adooq.com/y-27632-dihydrochloride.html) |
| A-83-01 | A | 0.5 uM | [Wako Pure Chemical Industries, 035-24113（10mg）](https://labchem-wako.fujifilm.com/jp/product/detail/039-24111.html) |
| CHIR99021 | C | 3 uM | [AdooQ BioScience, A10199-100](https://www.adooq.com/chir-99021.html) |
| BEC digestion medium | | | |
| Leibovitz L-15 medium | - | - | Gibco, 11415-064 |
| HEPES | - | 20 mM | Gibco, 15630-080 |
| Galactose | - | 1 g/L | Sigma, G0750-10G |
| L-proline | - | 30 mg/L | Sigma, P0380 |
| Insulin | - | 0.1 µM | Sigma-Aldrish, I9278-5ML |
| Dexamethasone Sodium Phosphate | Dex | 0.1 µM | Dexate injection, JAPAN |
| Antibiotic-Antimycotic | - | 1X | Gibco, 15240062 |
| BEC culture medium | | | |
| DMEM-LG |  | - | Wako, 041-29775 |
| Glucose |  | 3.5 g/L | Gibco |
| ITS supplement A | IST-A | 1X | Gibco, 51300-044 |
| Dexamethasone Sodium Phosphate | Dex | 0.1 µM | Dexate injection, JAPAN |
| Nicotinamide | NA | 10 mM | Sigma-Aldrish, N0636 |
| Ascorbic acid | Asc2P | 1 mM | Wako, 013-12061 |
| Recombinant epidermal growth factor | EGF | 10 ng/mL | [Sigma, E9644](https://www.sigmaaldrich.com/catalog/product/sigma/e9644?lang=ja&region=JP) |
| Recombinant hepatic rowth actor | HGF | 10 ng/ml | Peprotech, 100-39 |
| FBS | FBS | 10% | Gibco, 26140079 |
| Antibiotic-Antimycotic |  | 1X | Gibco, 15240062 |

**Table S2.** List of TaqMan primers used for qRT-PCR.

| Gene symbol | Gene name | TaqMan® ID | Amplicon Length |
| --- | --- | --- | --- |
| *Asbt (Slc10a2)* | Apical sodium-dependent bile acid transporter | Rn00691576_m1 | 148 |
| *Cftr* | Cystic fibrosis transmembrane conductance regulator | Rn01455970_m1 | 119 |
| *Ck19 (Krt19)* | Cytokeratin 19, type I | Rn01496867_m1 | 91 |
| *CK7 (Krt7)* | Cytokeratin 7, type II | Rn04224249_u1 | 73 |
| *Epcam* | Epithelial cell adhesion molecule | Rn01473202_m1 | 117 |
| *Gapdh* | Glyceraldehyde-3-phosphate dehydrogenase | Rn99999916_s1 | 87 |
| *Ggt1* | Gamma-glutamyltransferase 1 | Rn00587709_m1 | 91 |
| *Sox9* | SRY-box transcription factor 9 | Rn01751070_m1 | 102 |
| *Hnf1β* | HNF1 homeobox B | Rn00447453_m1 | 78 |
| *Ki67* | Marker of proliferation Ki-67 | Rn01451446_m1 | 104 |
| *Foxm1* | Forkhead box M1 | Rn00581221_m1 | 86 |
| *Pcna* | Proliferating cell nuclear antigen | Rn01514538_g1 | 94 |
| *Dik1* | Delta-like 1 homolog | Rn00587011_m1 | 74 |
| *Jag1* | Jagged canonical Notch ligand 1 | Rn00569647_m1 | 62 |
| *Notch1* | Notch receptor 1 | Rn01758633_m1 | 87 |
| *Notch2* | Notch receptor 2 | Rn01758633_m1 | 56 |
| *Notch 3* | Notch receptor 3 | Rn00571731_m1 | 112 |
| *Notch4* | Notch receptor 4 | Rn01525734_m1 | 74 |
| *Hes1* | Hes family bHLH transcription factor 1 | Rn00577566_m1 | 105 |
| *Hey1* | Hes-related family bHLH transcription factor with YRPW motif 1 | Rn00468865_m1 | 80 |
| *Hes5* | Hes family bHLH transcription factor 5 | Rn00821207_g1 | 103 |
| *Tgfβ1* | Τransforming growth factor, beta 1 | Rn99999016_m1 | 76 |
| *Tnfα* | Τumor necrosis factor α | Rn99999017_m1 | 108 |
